# Isolation after Diphtheria
1Read at the meeting of the Bristol Medico-Chirurgical Society on April 13, 1898.


**Published:** 1898-06

**Authors:** Bertram M. H. Rogers

**Affiliations:** Physician and Pathologist to the Royal Hospital for Sick Children and Women, Bristol


The Bristol

Medico-Chirurgical Journal.

JUNE, 1898.

### ISOLATION AFTER DIPHTHERIA.[1]

BY

BERTRAM M. H. ROGERS, B.A., M.D., B.Ch. Oxon.,

*Physician and Pathologist to the Royal Hospital for Sick Children and Women, Bristol.*

WHILST endeavouring to discover the cause of an outbreak of diphtheria in the girls' medical ward of the Children's Hospital, it occurred to me that it was within the bounds of possibility that the disease had been introduced by a patient, suffering from some other complaint, who had still active bacilli present in the throat or nose after some previous attack of diphtheria. This opinion was subsequently supported by the facts that came out on a systematic examination of all the cases in the ward and of cultures obtained during the apparent convalescent stage of that disease.

Before the introduction of the routine bacteriological examination by the Medical Officer of Health for the city of Bristol, most of us were, I believe, content to allow our diphtheria patients to go home or mix with other children at the end of a month, counting from the beginning of the illness, provided that no membrane could be seen in the throat and no nasal discharge existed. So universal has been this practice, that most if not all text-books state that isolation should be

[1] Read at the meeting of the Bristol Medico-Chirurgical Society on April 13, 1898.

enforced for only that period, while the Association of Medical Officers of Public Schools have fixed that limit before a boy may be allowed to return. I am quite prepared to admit that in the great majority of cases this period of time is quite sufficient, and that often it is a far longer time than is necessary; but the facts that I have collected go to prove that in some cases a much more protracted period of isolation is necessary and must be enforced.

In diphtheria we have a disease that is year by year becoming more common—a fact which I believe is generally accepted,—and so little is known, in spite of the vast collections of statistics on the subject, of how the disease is spread, that any suggestion, such as the one to be gathered from this paper, must be acceptable, and the practical conclusions to be learnt are of importance to physicians whether in practice or engaged in public health work.

It has been my practice at the Children's Hospital, ever since our Medical Officer of Health undertook to examine the swabs, to have inoculated tubes sent at short intervals for examination, and no patient has been allowed to leave the diphtheria ward till I got a negative report. The question, then, very naturally rises in one's mind: Of what value is a negative report by itself? Does it mean that the throat and nose are free from bacilli? I will not go so far as to say that; for on one occasion a negative report was given me, with a request that a further specimen might be sent. On this being complied with, a positive one was returned. But in most cases I believe a negative report is reliable, particularly in those cases in which it is most important—*i.e.* those in which the bacilli have been found to be present for a considerable time. In such cases there is a distinct difference in the appearance of the bacilli; they take on a different form, known as the "raisin-seed" or "grape-seed" shape, which is now recognised as a degenerate form of the bacillus. An illustration of this change is given in Pearmain and Moor's *Applied Bacteriology.*

Unless some precautions such as these are taken, I believe many cases are dismissed long before the throat or nose is clear, and children with active, virulent bacilli in their respiratory passages are set free on the public. I can hardly imagine a better method of dissemination.

The outbreak to which I referred at the beginning of my paper had its counterpart at the General Hospital only a short time ago, and I hope someone who is better acquainted with the details will relate the experience there.

The first victim in our epidemic was a patient suffering from enteric fever. She was one of a family of three children who came under Dr. Elliott's care, and of these two died of diphtheria, contracted in the ward. She, when delirious in enteric, developed a foul nasal discharge, which was shown to contain D. bacilli. She died on November 7th. The second child early in November being convalescent, the temperature having been normal for a week again became febrile. This was at first taken for a relapse; but a nasal discharge appearing, followed by the appearance of membrane in the larynx necessitating tracheotomy, and a positive report being returned, proved the fever to be due to diphtheria. To this attack she succumbed.

The third child passed safely through both diseases. Following this, several other cases broke out. From a culture from Caroline V., admitted for chorea in September, a positive report was returned on December 5, a negative on January 4, 1898; from Isabella B. a positive was got on December 15, a negative on January 4; from Annie G. a positive on December 2, a negative fourteen days later; while from a ward almost as remote as possible, Emily S., a child of seven weeks old, admitted for a surgical operation, developed nasal diphtheria on December 5.

I never discovered to my complete satisfaction the primary case of this small epidemic, but I suspect very strongly that it was a girl who was only in the ward for twenty-four hours, and was removed by her parents. She had, it is true, no obvious symptoms of diphtheria, but I hold that it is in these cases that the danger lies.

In one or two of the above cases the diagnosis was made solely on the results of the bacteriological examination; and as no case was observed before the beginning of November, and yet a child who had been in the ward for a month for chorea developed it, we may conclude that the disease was introduced from without and was not due to faulty drains—the usual assignable cause of an outbreak.

I have searched medical literature for some records of bacteriological examinations conducted systematically to discover how long the bacillus may remain active in the throat. Dr. Hermann Biggs,[1] of New York, examined 405 cases with this object in view, and in 245 no bacilli were found to be present three days after the disappearance of the membrane; so that in over 50 per cent. a very short period of isolation is required.

| | | | | | |
|---|---|---|---|---|---|
| But in | 103 | they could be found | for | 7 | days |
| ,, | 34 | ,, | ,, | 12 | ,, |
| ,, | 16 | ,, | ,, | 15 | ,, |
| ,, | 4 | ,, | ,, | 21 | ,, |
| and in | 3 | ,, | ,, | 35 | ,, |

Allowing, then, a week for the membrane to be present, at least 5 cases were dangerous for 41 days.

Observations of a similar character, but for a different object, were conducted by Dr. White.[2] He was testing the value of antiseptic sprays, etc., on the vitality of the bacillus, and in his observations the extreme limit was found to be, in spite of the attempts to destroy the parasite with a peroxide of hydrogen preparation known as pyrozone, 33 days from the commencement of the disease; whilst in one laryngeal case the bacilli were found 40 days after admission, and in another 48 days after the disappearance of the membrane.

Abel[3] has recorded a case in which for 65 days after the disappearance of the membrane the bacillus could be cultivated, and Dr. Macgregor,[4] in an article that appeared subsequent to the preparation of this paper, gives a case in which active bacilli were found in December in the throat of a boy aged six months, the attack of diphtheria having been recovered from in July; and Schäfer[5] cites cases in which they were found 7½ months after the attack.

[1] *Med. Rec.*, 1894, xlvi. 323. [2] *Ibid.*, 545.

[3] *Centralbl. f. Bakteriol. u. Parasitenk.*, 1893, xiv. 756; see *Brit. M. J.*, 1894, i. Epitome, p. 16, and *Am. Year-Book Med. & Surg.* 1896, p. 993.

[4] *Lancet*, 1898, i. 716. [5] *Brit. M. J.*, 1895, i. 61.

Dr. Astley Gresswell[1] argues that in some persons the disease becomes chronic, and may recrudesce on exposure to damp or cold. I cannot help expressing the opinion, after reading this paper, that Dr. Gresswell's conclusions seem to me to be drawn rather from suppositions, or perhaps failures to find a satisfactory cause of the outbreak, than on well-ascertained facts. How or where patients take the disease is one of those mysteries yet to be solved; and, further, it is a well-known fact that the protection afforded by one attack is of short duration. Besides, in no case does it appear that a culture experiment or a microscopic examination was made; so that I think we may dismiss this argument as not proven.

The exceptional cases that have come under my observation are four. In each case antitoxin was given, and antiseptic sprays were employed in the nasal cases.

CASE 1.—Emily S., aged seven weeks, was admitted under Mr. Kendall for talipes, on September 4, 1897, and on December 1 it was discovered she had nasal diphtheria, the following reports being sent by the Medical Officer of Health :

Emily S., four months.

December 8, 1897.—Large amount and large club forms and wedges of diphtheria. Staphylococcus aureus. Diplococci.
December 24, 1897.—Diphtheria tending to involution forms. Diplococci. Staphylococcus aureus.
January 4, 1898.—Large amount and large wedge and involution forms of diphtheria. Staphylococcus aureus. Diplococci.
January 12, 1898.—Large amount, involution forms, wedges, and clubs of diphtheria. Staphylococci.
January 22, 1898.—Large amount, medium variety of diphtheria. Diplococci. Staphylococcus aureus.
February 1, 1898.—No diphtheria found. Staphylococcus albus. Diplococci.
February 3, 1898.—Fair amount, medium variety of diphtheria (well-marked wedges in violet, highly differentiated in blue). Staphylococcus aureus.
February 8, 1898.—Wedges and involution forms of diphtheria, fair amount. Diplococci.
February 18, 1898.—Wedges and involution forms of diphtheria, fair amount. Staphylococcus aureus. Diplococci.
February 26, 1898.—Luxuriant growth of diphtheria. Some staphylococci.
March 8, 1898.—Large amount, medium variety of diphtheria. Staphylococcus aureus.
March 18, 1898,—Large amount, medium variety (growth still luxuriant) of diphtheria. Staphylococcus aureus.
March 27, 1898.—No diphtheria found. Staphylococci. Diplococci.

[1] *Tr. Epidemiol. Soc. Lond.*, 1887, v. 57.

CASE 2.—Willie E., aged five, was admitted on June 17, 1897, with diphtheritic membrane on the fauces. Tracheotomy was done the following day by Mr. Kendall, and the patient progressed well, but unfortunately some stenosis of the trachea followed, producing inspiratory dyspnœa.

The following reports were given in this case:

June 18.—D. bacillus. Staphylococcus aureus.
June 27.—D. bacilli in large numbers. Staphylococci and small torulæ.
July 10.—D. bacilli still in good amount, some showing tendency to involution, but many apparently still in active growth.
July 27.—D. bacilli, active variety, has apparently assumed a chronic form.
August 14.—D. bacilli, marked numbers. Much staphylococcus aureus.
August 17.—D. bacilli still in large numbers, in a vigorous state of growth. Staphylococcus aureus. Torulæ.
August 24.—D. bacilli, large amount of medium. Staphylococcus aureus. Diplococci.
September 1.—(No D. bacilli.) A few suspicious forms, apparently involution staphylococci and diplococci, single and in chains, short streptococci.
September 3.—(No D. bacilli.) Staphylococci, diplococci, and short streptococci.

Therefore the bacilli were found from June 17 to August 24, a period of 68 days at the least, for the disease was present a day or two before the child came in, and the bacilli may have been in the throat after August 24 for a day or two.

The sequel of this case is interesting. The boy was admitted again in December, when the stenosis of the trachea nearly caused his death by asphyxia. Traction on the tongue brought about instant relief, and, no further attacks following, he was discharged. On February 7 I was summoned to attend his inquest. He had been found dead in bed, deeply cyanosed. No doubt another attack of dyspnœa had come on, and having no one to relieve him he died. The coroner did not accede to my request for a necropsy, so I cannot state positively the condition of the trachea.

CASE 3.—For the reports on this case I am indebted to Dr. Davies.

Mildred C., aged thirty-two, was notified on January 11, 1897, and a positive report given the next day.
January 13.—D. bacilli very small. Staphylococcus albus. Diplococci and torulæ.
January 19.—D. bacilli, moderate amount. Some involution forms. Diplococci and streptococci.

January 23.—D. bacilli, some small groups of typical wedges and diplococci chains. Fat bacilli in chains. Staphylococci.
February 2.—D. bacilli. One well-marked group. Diplococci invading epithelial cells. Not yet free.
February 12.—D. bacilli. Growth not very typical. Some large staphylococci colonies and fine growth. Still some involution forms of D. bacilli left.
February 20.—D. bacilli absent.

This gives a period of 32 days.

In addition to these cases, I have another, Winifred B., aged four, in whom the bacillus was found for 28 days—a nasal case; and another, Edith C., aged twenty-nine, for 23 days; in each of these the dates being taken from the first examination, and not from when the disease was first noticed.

The lesson that is to be learnt from these cases seems to me to be that no case should be allowed to mix with healthy persons until bacteriological examination has shown that the throat and nares are free from the bacillus, instead of mere isolation till the membrane appears to have gone. I believe that if this were more rigidly adhered to we should check the spread of diphtheria, which now appears in a most inexplicable way.

---

In the discussion which followed the reading of Dr. Rogers's paper, Dr. Michell Clarke emphasised the importance of the subject dealt with by Dr. Rogers, and gave an account of a small outbreak of diphtheria at the General Hospital last year, which appeared to have originated from a child who suffered from a slight purulent nasal discharge, but had not had diphtheria. He alluded to the case of another child, in whose throat the diphtheria bacilli persisted for a very long time after the membrane had disappeared.—Dr. Watson Williams drew attention to the need of recognising the nose as a possible source of the infection when diphtheria arose without obvious origin. It was now usual to make swab cultures out of the throat, but it was also important to examine the nasal passages and even, in some cases, to make cultures before we could safely declare a patient free from infection. He drew attention to an exhaustive article by Dr. Heaven, read before the Medical Officers of Health Association, dealing with the question of fibrinous rhinitis as a source of diphtherial infection.—Dr. J. O. Symes had made frequent examinations in nine cases, and found the bacillus to persist, until the twelfth week, 12, 13, 20, 30, 22, 35, 32, and 24 days—an average of 30 days. He remarked that after the first ten days or fortnight the bacillus became altered in character, being short and "sprig-like." This short bacillus was not pathogenic to guinea-pigs. It frequently persisted for many weeks. Was this a modified Klebs-Löffler bacillus or the pseudo-bacillus? and were patients to be isolated when this variety persisted?—Dr. Markham Skerritt said that it had been stated that the disease did not necessarily spread to those brought into contact with a still infectious patient; but that the closer this contact, the greater the probability of infection. He pointed out that this was not peculiar to diphtheria, and gave a striking instance where scarlet fever did not extend under conditions apparently most favourable to its dissemination. Hence it could not be assumed, because diphtheria failed to spread from a convalescent patient in whom the bacillus was still present, that therefore the organism was no longer in a virulent stage.—Dr. SHINGLETON SMITH, speaking on behalf of the much-tried and long-suffering parents of the boys in our public schools, hoped that the medical officers would not impose a long and probably unnecessary quarantine in all cases which were called diphtheria, in which the contagion appeared to be easily controlled and the risk of an extension of the disease infinitesimal. It did not follow that because an attenuated bacillus still existed, that therefore the patient should still be dangerous to those around him, and all clinical evidence tended to show that the degree of contagion could not be in proportion to the presence of the bacilli which had been found to survive for so many months after an attack.—In reply, Dr. ROGERS stated that he could not agree with Dr. Symes's view that our present knowledge of the bacteriology of diphtheria was very incomplete, nor did he think that the prolonged presence of the bacillus could be accounted for by a confounding of the pseudo-bacillus with the Klebs-Löffler. He referred to the work of Hewlett and Knight, who claim that they have been able to convert the pseudo-bacillus into the Klebs-Löffler by careful regulation of the temperature as well as the serum process; and also to Martin's investigations, which tended to show that the pseudo-bacillus can give rise to severe and genuine diphtheria if associated with streptococci, a fact which had also been made clear by Hewlett and Knight. So that even if the pseudo-bacillus was the one observed, it might, and often apparently did, cause diphtheria. He thought that the differentiation of the two was founded on very slight evidence.

